# Progress and Decline of the Epidemic

**Published:** 1849-07

**Authors:** 


### PROGRESS AND DECLINE OF THE EPIDEMIC.

The following statements show the course of the cholera in this city, from the 10th of May, when it first became epidemical:

| | |
|---|---|
| From May 10th to June 15th, | 75 |
| " June 16th to July 16th | 2475 |
| " July 17th to July 23d | 293 |
| " July 24th to July 30th | 107 |
| Aggregate of deaths by cholera, | 2950 |

The entire mortality of the city for the period of six weeks, from all diseases, is shown by the following weekly aggregates:

| | |
|---|---|
| Week ending June 25th | 568 |
| Week ending July 2d | 940 |
| Week ending July 9th | 1022 |
| Week ending July 16th | 950 |
| Week ending July 23d | 512 |
| Week ending July 30th | 285 |
| Total mortality for six weeks | 4277 |

The first decided abatement in the ravages of the epidemic, and improvement in the general health of the city, was shown by the report of July 15. Since that time the daily reports have been as represented below:

| | Chol. | O. D. | Total. |
|---|---|---|---|
| July 15th | 87 | 67 | 154 |
| " 16th | 60 | 44 | 104 |
| " 17th | 61 | 40 | 101 |
| " 18th | 59 | 40 | 99 |
| " 19th | 43 | 29 | 72 |
| " 20th | 32 | 28 | 60 |
| " 21st | 36 | 31 | 67 |
| " 22d | 33 | 25 | 58 |
| " 23rd | 29 | 26 | 55 |
| " 24th | 20 | 20 | 40 |
| " 25th | 19 | 25 | 44 |
| " 26th | 15 | 31 | 46 |
| " 27th | 17 | 29 | 46 |
| " 28th | 13 | 25 | 38 |
| " 29th | 14 | 23 | 37 |
| " 30th | 9 | 25 | 34 |
| Aug. 1st | 5 | 16 | 21 |
| " 2d | 13 | 20 | 33 |
| " 3d | 10 | 16 | 26 |
| " 4th | 11 | 23 | 34 |
| " 5th | 12 | 20 | 32 |
| " 6th | 6 | 23 | 29 |

The epidemic has so nearly disappeared, that the Board of Health have determined to meet and report but three times a week.

